# Supplementary material for: Overseas reception of English translations of Journey to the West: Temporal dynamics, cross-platform sentiment patterns, and topic modeling
Source: PLoS One. 2026 Apr 21;21(4):e0347253. doi: 10.1371/journal.pone.0347253 (PMC13098970; doi:10.1371/journal.pone.0347253)
Supplement: Attachment — Submitted filename: Response to Reviewers.docx [file pone.0347253.s003.docx]

Dear Editors and Reviewers,

We wish to express our sincere gratitude for the opportunity to revise our manuscript entitled “Overseas reception of English translations of *Journey to the West*: Temporal dynamics, cross-platform sentiment patterns, and topic modeling” (ID: PONE-D-25-41113). We deeply appreciate the editorial guidance and the reviewers’ insightful comments, which have been instrumental in improving the methodological rigor, analytical depth, and clarity of exposition in our work.

We have thoroughly revised the manuscript to address all points raised. Below are our point-by-point responses to the Editors’ and Reviewers’ comments, with the location of each change specified in the revised manuscript.

Notably, we have provided a direct comparison between the original and revised texts for key changes to facilitate your review. For clarity, the reviewers’ and editors’ original comments are reproduced in full and highlighted in blue to distinguish them from our responses.

Best regards.

**Response to Academic Editors**	2

Comment 1	2

Comment 2	2

Comment 3	3

Comment 4	4

Comment 5	5

Comment 6	5

**Response to Reviewer #1**	6

Comment	6

**Response to Reviewer #2**	10

Comment 1	10

Comment 2	14

Comment 3	22

**Response to Reviewer #3**	26

Comment	26

**Response to Academic Editors**

We sincerely thank the Academic Editors for providing the opportunity to revise it. We also appreciate the comprehensive list of journal requirements, which has guided us in ensuring our manuscript fully complies with PLOS ONE’s policies. We have addressed all points below and believe the manuscript is now much stronger.

**Comment 1:** Please ensure that your manuscript meets PLOS ONE's style requirements, including those for file naming. The PLOS ONE style templates can be found at

https://journals.plos.org/plosone/s/file?id=wjVg/PLOSOne_formatting_sample_main_body.pdf and<https://journals.plos.org/plosone/s/file?id=ba62/PLOSOne_formatting_sample_title_authors_affiliations.pdf>

**Response**: We thank the editor for this reminder. We have carefully verified and ensured that the manuscript complies with the PLOS ONE style templates. These checks covered all elements specified in the journal’s guidelines, including the document structure and headings, formatting for figures and tables (along with their labels and legends), the reference list and in-text citations, and additional formatting details such as font, spacing, margins, and file naming.

**Comment 2**: In your Methods section, please include additional information about your dataset and ensure that you have included a statement specifying whether the collection and analysis method complied with the terms and conditions for the source of the data.

**Response**: We apologize for the earlier oversight, and thank the editor for this guidance.

Regarding the compliance statement: We have revised the Methods section to explicitly confirm compliance with the platforms’ terms. This change is in the revised manuscript (lines 220-225, page 11) and presented below for your convenience.

Regarding the additional dataset information: We have already included dataset details in Section Data and data processing, covering the data sources, initial collection volume, final validated corpus, preprocessing procedures and edition-related data. For verification, please refer to pages 9-11 of the revised manuscript.

Comparison of Original and Revised Text

*Note: In the ‘Original’ column, strikethrough text indicates content that has been removed in the revised manuscript. In the ‘Revised’ column, text highlighted in red indicates new additions in the revised manuscript.*

| **Original** | **Revised** |
| --- | --- |
| ~~The dataset was acquired through Python-based web scraping, capturing review timestamps, anonymized user identifiers, and textual content while preserving source platform metadata (Amazon/Goodreads) and translator attribution.~~ The collected reviews underwent standard text preprocessing, including removal of HTML tags, URLs, and non-alphanumeric symbols, with whitespace normalization and case unification. Non-English entries (notably Spanish-language reviews prevalent on Goodreads) and duplicate or non-substantive comments were systematically excluded, resulting in a final corpus of 1,795 validated English reviews for analysis. | Our data collection and analysis were conducted in compliance with Amazon’s and Goodreads’ terms of service, which permit non-commercial academic study. The reviews were collected via Python-based web scraping from publicly accessible pages, consistent with established practices in computational literary studies [35]. To protect contributor privacy, all reviews were anonymized upon collection, in accordance with principles outlined in ethical guidelines for internet research [36]. The collected reviews underwent standard text preprocessing, including removal of HTML tags, URLs, and non-alphanumeric symbols, with whitespace normalization and case unification. Non-English entries (notably Spanish-language reviews prevalent on Goodreads) and duplicate or non-substantive comments were systematically excluded, resulting in a final corpus of 1,795 validated English reviews for analysis. |

Full references for the citations added in the revised text are provided below.

35.	Parnell C, Driscoll B. Institutions, platforms and the production of debut success in contemporary book culture. Media International Australia. 2023;187(1): 123–138. doi: 10.1177/1329878X211036192

36.	Heise AHH, Hongladarom S, Jobin A, Kinder-Kurlanda K, Sun S, Lim EL, et al. Internet research: Ethical guidelines 3.0. WildApricot. 2019 [cited 2025 November 18]. Available: https://members.aoir.org/resources/Documents/IRE%203.0%20-%20final%20distribution%20copy%20for%20AoIR%20members.pdf

**Comment 3**: Please note that PLOS One has specific guidelines on code sharing for submissions in which author-generated code underpins the findings in the manuscript. In these cases, we expect all author-generated code to be made available without restrictions upon publication of the work. Please review our guidelines at https://journals.plos.org/plosone/s/materials-and-software-sharing#loc-sharing-code and ensure that your code is shared in a way that follows best practice and facilitates reproducibility and reuse.

**Response:** We thank the editor for highlighting this important policy. We confirm that our manuscript fully complies with PLOS ONE's code sharing guidelines. All author-generated codes supporting the findings of this study, including the sentiment analysis and LDA topic modeling scripts, have been made publicly available without restrictions. The codes, along with the custom domain sentiment lexicon, are hosted in a GitHub repository (<https://github.com/Dandanjun518/JTTW_Overseas_Reception_Analysis>) and have been permanently archived on Zenodo under DOI 10.5281/zenodo.17735145 to ensure long-term accessibility. The repository is licensed under the MIT license, which permits open reuse. This information is clearly stated in the Data Availability Statement.

**Comment 4:** We note that Figure 1 in your submission contain copyrighted images. All PLOS content is published under the Creative Commons Attribution License (CC BY 4.0), which means that the manuscript, images, and Supporting Information files will be freely available online, and any third party is permitted to access, download, copy, distribute, and use these materials in any way, even commercially, with proper attribution. For more information, see our copyright guidelines: <http://journals.plos.org/plosone/s/licenses-and-copyright>.

 We require you to either (1) present written permission from the copyright holder to publish these figures specifically under the CC BY 4.0 license, or (2) remove the figures from your submission:

 1. You may seek permission from the original copyright holder of Figure 1 to publish the content specifically under the CC BY 4.0 license.

We recommend that you contact the original copyright holder with the Content Permission Form (http://journals.plos.org/plosone/s/file?id=7c09/content-permission-form.pdf) and the following text:

“I request permission for the open-access journal PLOS ONE to publish XXX under the Creative Commons Attribution License (CCAL) CC BY 4.0 (http://creativecommons.org/licenses/by/4.0/). Please be aware that this license allows unrestricted use and distribution, even commercially, by third parties. Please reply and provide explicit written permission to publish XXX under a CC BY license and complete the attached form.”

Please upload the completed Content Permission Form or other proof of granted permissions as an "Other" file with your submission.

 In the figure caption of the copyrighted figure, please include the following text: “Reprinted from [ref] under a CC BY license, with permission from [name of publisher], original copyright [original copyright year].”

 2. If you are unable to obtain permission from the original copyright holder to publish these figures under the CC BY 4.0 license or if the copyright holder’s requirements are incompatible with the CC BY 4.0 license, please either i) remove the figure or ii) supply a replacement figure that complies with the CC BY 4.0 license. Please check copyright information on all replacement figures and update the figure caption with source information. If applicable, please specify in the figure caption text when a figure is similar but not identical to the original image and is therefore for illustrative purposes only.

**Response:** We apologize for the oversight in our previous submission and thank the editor for the clarification. We were unable to obtain permission from the copyright holders (Amazon and Goodreads) to publish their logos under the CC BY 4.0 license. Therefore, we have replaced the figure with a new, fully original one. The revised figure uses the generic term "online reviews" and thus avoids all proprietary names and logos. The updated figure is provided below and has been uploaded to the submission system.

**Revised:**

**Comment 5**: We are unable to open your Supporting Information file S1_Code.py and S2_Code.py. Please kindly revise as necessary and re-upload.

**Response:** We apologize for the technical issue with the Supporting Information files. To ensure the long-term accessibility and stability of the code, we have deposited codes in a public GitHub repository (<https://github.com/Dandanjun518/JTTW_Overseas_Reception_Analysis>) and has been permanently archived on Zenodo (10.5281/zenodo.17735145). The repository is licensed under the MIT license, allowing for unrestricted reuse. This information is clearly stated in the Data Availability Statement. Therefore, we have removed the code files from the Supporting Information section to avoid confusion.

**Comment 6:** We note that there is identifying data in the Supporting Information file “S1_Data.xlsx”. Due to the inclusion of these potentially identifying data, we have removed this file from your file inventory. Prior to sharing human research participant data, authors should consult with an ethics committee to ensure data are shared in accordance with participant consent and all applicable local laws.

Data sharing should never compromise participant privacy. It is therefore not appropriate to publicly share personally identifiable data on human research participants. The following are examples of data that should not be shared:

-Name, initials, physical address

-Ages more specific than whole numbers

-Internet protocol (IP) address

-Specific dates (birth dates, death dates, examination dates, etc.)

-Contact information such as phone number or email address

-Location data

-ID numbers that seem specific (long numbers, include initials, titled “Hospital ID”) rather than random (small numbers in numerical order)

Data that are not directly identifying may also be inappropriate to share, as in combination they can become identifying. For example, data collected from a small group of participants, vulnerable populations, or private groups should not be shared if they involve indirect identifiers (such as sex, ethnicity, location, etc.) that may risk the identification of study participants.

Additional guidance on preparing raw data for publication can be found in our Data Policy (https://journals.plos.org/plosone/s/data-availability#loc-human-research-participant-data-and-other-sensitive-data) and in the following article: <http://www.bmj.com/content/340/bmj.c181.long>.

Please remove or anonymize all personal information (<specific identifying information in file to be removed>), ensure that the data shared are in accordance with participant consent, and re-upload a fully anonymized data set. Please note that spreadsheet columns with personal information must be removed and not hidden as all hidden columns will appear in the published file.

**Response:** We sincerely apologize for this oversight and thank the editor for identifying it. We have taken immediate action to address this critical issue. In compliance with the platforms' Terms, we have made the decision not to share the reviews. Instead, we provide a new, fully de-identified dataset as Supporting Information S1 File. This new dataset contains only the anonymized review ID, year, platform, translator and the analytical results (including sentiment scores, sentiment label, dominant topic, and topic probabilities), with all reviews removed. We have verified that the new file contains no hidden columns or rows, and have meticulously checked all content to ensure no personal information remains. We believe this approach ensures the reproducibility of our research while respecting the spirit of data protection and platform governance.

**Response to Reviewer #1**

We sincerely thank the reviewer for the constructive comments, which have helped us improve the clarity and focus of our manuscript. We have carefully considered the comments and have revised the abstract and conclusion sections accordingly. Our point-by-point responses are detailed below.

**Comment:** The conclusion section fails to provide a sufficient summary of the entire text and deviates to some extent from the original title. Some expressions are not authentic enough. For example, the abstract part could be slightly modified.

**Response:** We thank the reviewer for this important feedback.

First, in Conclusion Section**,** we have rewritten the conclusion to provide a more comprehensive summary of the study’s findings. Additionally, a paragraph which was deemed to deviate from the title has been removed. The changes have been made in the revised manuscript (lines 686-752, pp. 33-36).

Comparison of Original and Revised Text

*Note: In the ‘Original’ column, strikethrough text indicates content that has been removed in the revised manuscript. In the ‘Revised’ column, text highlighted in red indicates key changes in the revised manuscript.*

| **Original** | **Revised** |
| --- | --- |
| By analyzing online reader reviews from Amazon and Goodreads, this study pioneers a tripartite analytical framework, encompassing temporal dynamics, cross-platform sentiment patterns, and topic modeling, to investigate the overseas reception of four renowned *JTTW* translations by Waley, Yu, Lovell, and Jenner.  Temporal analysis of reviews reveals two distinct patterns, universal growth in overall review counts as well as divergent trajectories among individual translators. The former is driven by surging global interest in *JTTW* catalyzed by multimedia adaptations while the latter is attributable primarily to variations in their patronage systems and accumulated cultural capital. ~~A key methodological innovation lies in the hybrid sentiment lexicon integrating domain sentiment lexicon with VADER-NRC-AFINN through weighting mechanism.~~ Sentiment analysis revealed predominantly positive sentiment toward overall reviews, yet exposed significant cross-platform divergences. These divergences originate from platforms’ distinct evaluative mechanisms. Within Amazon’s e-commerce framework, its broader and diversified user base positions evaluations of translated works primarily within transactional aspects. Conversely, Goodreads’ social reading ecosystem centers on literary content analysis, driven by its core readership community. Yu’s translation exhibited a marked cross-platform variation, a Bourdieusian dynamic of cultural capital valuation reshaped by digital field infrastructures. Complementing this approach, the LDA model enabled data-driven topic extraction, objectively identifying three core reader-focus dimensions. In assessing translation quality, Yu’s extensively annotated translations, valued as indispensable for contextual comprehension, achieved the highest positive reception, followed by Waley’s and Lovell’s versions. Notably, plot reception exhibited significantly heightened negativity across all translators, attributed to both narrative abridgment disappointing media-adapted readers and cross-cultural challenges posed by faithful renditions of the zhanghui structure and Buddhist hermeneutic frameworks. Regarding character portrayal, Lovell’s vivid characterizations and Yu’s poetic-philosophical retention surpassed Waley’s and Jenner’s approaches in reader preference.    Beyond documenting empirical findings, this study advances methodological discourse by integrating computational tools into humanistic frameworks. ~~Positioning sentiment analysis and topic modeling as interpretive scaffolds rather than technical endpoints, it transcends quantitative metrics to excavate cultural dynamics. Within this approach, quantitative patterns reveal negotiated meanings inherent to cross-cultural reception processes. The domain sentiment lexicon and LDA model function as hermeneutic aids, elucidating the platform-reader-text interplay. This approach aligns with humanistic epistemology that prioritizes contextual resonance over statistical precision [66]. Crucially, these tools derive validity through their capacity to activate dialogic interpretation—a process consonant with translation studies’ framework of meaning co-construction [67].~~  The study’s scope is limited by its reliance on two platforms, and the domain sentiment lexicon needs ongoing refinement to better capture how readers’ narrative interpretations are influenced. Future research could broaden its cultural scope by incorporating more platforms and diversifying dataset scales, while refining the domain sentiment lexicon through joint annotation efforts to deepen its adaptability to literary translations. Such advancements would enrich our grasp of how translated literature negotiates meaning across digital cultural landscapes. | By analyzing online reader reviews from Amazon and Goodreads, this study has delineated the complex reception landscape of English translations of *Journey to the West* through a tripartite analytical framework of temporal dynamics, cross-platform sentiment patterns, and topic modeling. With a hybrid sentiment lexicon as a key methodological innovation, the study delivers three principal contributions to the study of translation reception in digital environments.  First, the research elucidates the compound drivers of reader engagement over time. While a universal increase in review counts is propelled by surging global interest in *JTTW* multimedia adaptations, the divergent trajectories of individual translators are primarily attributable to the specific patronage systems and accumulated cultural capital surrounding their works. Second, the analysis demonstrates that the digital platforms act as agents in reconfiguring literary valuation. The significant sentiment divergence between Amazon’s e-commerce model, which prioritizes transactional utility, and Goodreads’ social-literary ecosystem, centered on discursive engagement, reveals that evaluative mechanisms are deeply embedded in platform architecture. The case of Yu’s translation, which exhibited a marked cross-platform variation in reception, exemplifies a Bourdieusian dynamic of cultural capital valuation reshaped by digital field infrastructures. Finally, the identification of three core reader concerns, namely, translation quality, plot acceptance, and character portrayal, pinpoints the specific loci of cross-cultural negotiation. Yu’s extensively annotated translations, valued as indispensable for contextual comprehension, achieved the highest positive reception for quality. Notably, plot reception exhibited significantly heightened negativity across all translators, highlighting the enduring tension between cultural fidelity and reader accessibility as a central challenge in literary translation. Regarding character portrayal, Lovell’s vivid characterizations and Yu’s poetic-philosophical retention surpassed Waley’s and Jenner’s approaches in reader preference.  By integrating computational tools as interpretive scaffolds, this study transcends quantitative metrics to reveal cultural dynamics, demonstrating the value of such methods for digital humanities scholarship. Admittedly, the study’s scope is limited by its reliance on two platforms, and the domain sentiment lexicon needs ongoing refinement to better capture how readers’ narrative interpretations are influenced. Future research could broaden its cultural scope by incorporating more platforms and diversifying dataset scales, while refining the domain sentiment lexicon through joint annotation efforts to deepen its adaptability to literary translations. Such advancements would enrich our grasp of how translated literature negotiates meaning across digital cultural landscapes. |

Second, in Abstract Section, we have polished and refined the language throughout to enhance its academic tone and clarity. The changes have been made in the revised manuscript (lines 23-51, pp. 2-3).

| **Original** | **Revised** |
| --- | --- |
| This study investigates the overseas reception of English translations of *Journey to the West* through temporal dynamics, cross-platform sentiment patterns, and topic modeling of 1,795 validated reviews from Amazon and Goodreads, covering four celebrated translators: Arthur Waley, Antony C. Yu, Julia Lovell, and W.J.F. Jenner.  Methodologically, a hybrid sentiment lexicon was developed by integrating a domain sentiment lexicon with AFINN, NRC, and VADER through weighted fusion, addressing the limited adaptability of general sentiment lexicons in translated literature analysis. Complementarily, LDA modeling was applied to enable data-driven theme extraction, ensuring objectivity in identifying reader discourse patterns.  Key findings reveal a consistent year-on-year increase in review counts across all translations, with Lovell’s version demonstrating the most rapid growth. While overall sentiment toward the translations is predominantly positive, significant cross-platform patterns emerge, yielding differential reception across translators. These patterns reflect the divergent evaluative mechanisms of digital platforms. Thematic analysis identifies three reader-focus themes: translation quality, plot acceptance, and character portrayal, with plot acceptance showing notably higher negativity compared to the other themes. Translator-level analysis reveals differential performance across themes, though not statistically significant.  This study advances cross-cultural reception theory by revealing how digital platforms reconfigure literary translation valuation, and pioneers a methodological framework capturing the dynamic interplay between reader perception, media infrastructure, and textual mobility, offering novel pathways for translation-digital humanities research. | The overseas reception of classical literature through online platforms presents a critical lens for understanding cross-cultural dynamics in the digital age.  This study investigates the overseas reception of English translations of *Journey to the West* by analyzing a corpus of 1,795 reviews from Amazon and Goodreads to examine temporal dynamics, cross-platform sentiment patterns, and topic modeling. The analysis covers four celebrated translators: Arthur Waley, Antony C. Yu, Julia Lovell, and W.J.F. Jenner.  Methodologically, we developed a hybrid sentiment lexicon by integrating a domain sentiment lexicon with AFINN, NRC, and VADER through a weighted fusion, addressing the limited adaptability of general sentiment lexicons in translated literature analysis. LDA modeling was further applied to enable data-driven theme extraction.  Key findings reveal a consistent year-on-year increase in review counts across all translations. Notably, despite an overall positive sentiment, significant cross-platform divergences emerge, reflecting the distinct evaluative mechanisms of digital platforms. Thematic analysis identifies three central reader concerns: translation quality, plot acceptance, and character portrayal, with plot acceptance exhibiting markedly higher negativity. Furthermore, translator-level analysis reveals performance variations across these themes.  This study demonstrates how digital platforms reconfigure valuation of literary translation, and pioneers a methodological framework for capturing the dynamic interplay between reader perception, media infrastructure, and textual mobility, offering new pathways for digital humanities research in translation studies. |

**Response to Reviewer #2**

We are deeply grateful to the reviewer for the exceptionally thorough, constructive, and insightful comments. The detailed suggestions have been invaluable in guiding our revisions and have significantly strengthened the manuscript.

**Comment 1**: This manuscript presents an engaging and methodologically sophisticated study on the overseas reception of Journey to the West translations. Its principal strength lies in its genuine interdisciplinary approach, integrating problems and theories in translation studies with advanced digital DH methods (e.g., hybrid sentiment analysis, LDA topic modeling). The research questions are well-defined, the analysis is robust, and the discussion provides deep interpretations that move beyond quantitative description. The construction of a domain-specific sentiment lexicon is a particularly notable methodological innovation.

While the manuscript is strong and potentially publishable a, I have several suggestions:

1. Strengthening the Literature Review and Theoretical Framing
The introduction effectively establishes the context and importance of the research. However, the review of computational methods could be updated and more tightly linked to the theoretical framework. The review of sentiment analysis and, in particular, deep learning methods (e.g., citing Tul et al. 2017) relies on literature that is becoming dated. The field of NLP has evolved rapidly with the advent of transformer-based models (e.g., BERT, GPT). Furthermore, the connection between the theoretical foundations (e.g., Reader-Response theory) and the chosen computational methods, while present, could be more explicit.

Suggestion:
Incorporate more recent reviews or studies (e.g., from 2020 onwards) that discuss the application of contextualized embeddings (BERT, etc.) and Large Language Models (LLMs) in sentiment analysis and literary text analysis. This will demonstrate a command of the state-of-the-art and provide a stronger justification for the choice of a lexicon-based approach (i.e., its interpretability and efficiency for this specific task) over more complex, data-hungry models.

Also, add a brief paragraph that more explicitly bridge the theoretical concepts with the methods. For example, clearly state how sentiment analysis operationalizes Nida’s “equivalent response,” and how topic modeling captures the “dynamic process of meaning construction” central to Reader-Response theory.

**Response:** We thank the reviewer for constructive suggestions. To address it, we have updated the literature by removing dated references (e.g., Tul et al., 2017) and incorporating seven recent studies (from 2020 onwards) on transformer-based models and LLMs. The changes have been made in the revised manuscript (lines 90-113, pp. 5-6).

Comparison of Original and Revised Text

*Note: In the ‘Original’ column, strikethrough text indicates content that has been removed in the revised manuscript. In the ‘Revised’ column, text highlighted in red indicates new additions in the revised manuscript.*

| **Original** | **Revised** |
| --- | --- |
| Sentiment analysis (SA), the computational detection of emotional tones in text, has gained prominence in literary translation research as a tool to analyzes reader’s opinions, sentiments, appraisals, attitudes, and emotions [9]. The application of SA in translated literature represents a burgeoning field, with scholars increasingly using it to interrogate sentiment polarity in cross-lingual contexts [10]. ~~Numerous studies have harnessed TextBlob’s integrated components, including its Naive Bayes classifiers and Pattern Analyzer, alongside general sentiment lexicons like VADER for sentiment analysis of translated literature reviews [11]. Nevertheless, these approaches exhibit limitations in handling literary texts’ complex semantics, rendering them increasingly outdated [12]. Although deep learning networks offer advanced alternatives, their substantial data requirements and training costs present practical barriers [13].~~ | Sentiment analysis (SA), the computational detection of emotional tones in text, has gained prominence in literary translation research as a tool to analyzes reader’s opinions, sentiments, appraisals, attitudes, and emotions [9]. The application of SA in translated literature represents a burgeoning field, with scholars increasingly using it to interrogate sentiment polarity in cross-lingual contexts [10]. Current SA methodologies primarily fall into two categories: deep learning models and lexicon-based approaches. Deep learning models, particularly transformer-based models like BERT and large language models (LLMs), face significant hurdles when applied to niche domains such as reviews of classical literary translations. A primary constraint is their data-hungry nature. BERT require substantial data to perform reliably [11,12], a requirement that is equally, if not more, pronounced for LLMs [13,14]. This poses a particular challenge for specialized studies where sample sizes are often limited to a few thousand reviews or fewer. Compounding this issue is a domain adaptation gap. As these models are pre-trained on general corpora, they often exhibit weak recognition of nuanced, culture-specific expressions [15]. Furthermore, each model family has distinct shortcomings. For instance, the context window of BERT can hinder its ability to capture emotional correlations across long reviews [16], while LLMs are particularly prone to generating factually incorrect or hallucinated emotional interpretations [17]. Given these constraints, lexicon-based methods offer a compelling alternative due to their interpretability, lower computational cost, and efficacy with small datasets. |

The following references have been removed from the revised manuscript:

11.	Vinodini S. Analyzing Sentiments in Paulo Coelho’s Literary Works Using VADER Sentiment Analysis. 2023 International Conference on Innovative Computing, Intelligent Communication and Smart Electrical Systems (ICSES), Chennai, India: IEEE; 2023, pp. 1–6. doi: 10.1109/ICSES60034.2023.10465319.

12.	TAO Y, HU Y. A deep-learning-driven study on the reception of translated works: A case study of sentiment analysis for readers’ reviews on The Analects translated by D.C. Lau. Journal of Foreign Languages. 2024; 47:72–9.

13.	Tul Q, Ali M, Riaz A, Noureen A, Kamranz M, Hayat B, et al. Sentiment Analysis Using Deep Learning Techniques: A Review. Ijacsa. 2017;8. doi: 10.14569/IJACSA.2017.080657.

Full references for the citations added in the revised text are provided below.

11.	Mercha EM, Benbrahim H. Machine learning and deep learning for sentiment analysis across languages: A survey. Neurocomputing. 2023;531: 195–216. doi: 10.1016/j.neucom.2023.02.015

12.	Qi Y, Shabrina Z. Sentiment analysis using twitter data: A comparative application of lexicon- and machine-learning-based approach. Soc Netw Anal Min. 2023;13(1): 31. doi: 10.1007/s13278-023-01030-x

13.	Zhao WX, Zhou K, Li J, Tang T, Wang X, Hou Y, et al. A survey of large language models. arXiv.2303.18223 [Preprint]. 2025 [cited 2025 November 15]. Available from: http://arxiv.org/abs/2303.18223

14.	Raiaan MAK, Mukta MdSH, Fatema K, Fahad NM, Sakib S, Mim MMJ, et al. A review on large language models: Architectures, applications, taxonomies, open issues and challenges. IEEE Access. 2024;12: 26839–26874. doi: 10.1109/ACCESS.2024.3365742

15.	Sharma NA, Ali ABMS, Kabir MA. A review of sentiment analysis: Tasks, applications, and deep learning techniques. Int J Data Sci Anal. 2025;19(3): 351–388. doi: 10.1007/s41060-024-00594-x

16.	Abdullah T, Ahmet A. Deep learning in sentiment analysis: Recent architectures. ACM Comput Surv. 2022;55(8): 1–37. doi: 10.1145/3548772

17.	Yu L, Liu Q, Xiong D. LFED: A literary fiction evaluation dataset for large language models. arXiv.2405.10166 [Preprint]. 2024 [cited 2025 November 15]. Available from: http://arxiv.org/abs/2405.10166

Furthermore, as advised, we have added a new paragraph to explicitly bridge the sentiment analysis and topic modeling with the theoretical concepts. The changes have been made in the revised manuscript (lines 152-165, pp. 7-8).

| **Original** | **Revised** |
| --- | --- |
| …Building on established methodologies for thematic mining of reader reviews [12], this study extends LDA application to a more expansive corpus, seeking to uncover nuanced patterns of audience perception and interpretive trends in cross-cultural literary reception.  Based on the foregoing review, the present study intends to examine the overseas reception of English translations of *JTTW* from three perspectives: temporal dynamics, cross-platform sentiment patterns, and theme modeling. The following three research questions are to be addressed in this study. | …Building on established methodologies for thematic mining of reader reviews [31], this study extends LDA application to a more expansive corpus, seeking to uncover nuanced patterns of audience perception and interpretive trends in cross-cultural literary reception.  Given their capabilities, this study employs these computational methods to quantitatively investigate the core concerns of the theoretical frameworks outlined above. Specifically, sentiment analysis operationalizes the core concept of Nida’s Functional Equivalence theory by quantifying the valence and intensity of readers’ affective responses. This provides an empirical basis for evaluating the nature of the target-language readers’ emotional response, a key factor in assessing a translation’s functional equivalence. Simultaneously, LDA topic modeling offers a means to capture the dynamic, constructive nature of reception emphasized by Reader-Response Criticism. By inductively identifying the emergent themes that preoccupy readers, LDA reveals the collective hermeneutic processes through which meaning is negotiated and assigned to the translated work. Thus, the integration of sentiment analysis and topic modeling facilitates a large-scale, empirical inquiry into the reception process, effectively bridging theoretical postulation with data-driven analysis of how translated literature is actually received and interpreted by a global readership.    Based on the foregoing review, the present study intends to examine the overseas reception of English translations of *JTTW* from three perspectives: temporal dynamics, cross-platform sentiment patterns, and theme modeling. The following three research questions are to be addressed in this study. |

Comment 2: 2. Deepening the Discussion and Interpretation of Findings
The discussion is generally strong but has opportunities for more nuanced analysis and concision. The explanation for cross-platform differences in Section 3.2, while valid, is somewhat repetitive in attributing the cause to both platform mechanisms and user bases, which are linked. The discussion on plot acceptance (Section 3.3.3) leans slightly towards attributing negative sentiment to reader “misinterpretation,” which could be balanced with a more translator-centered perspective. Finally, the analysis of Figure 9 (translator-by-theme sentiment) focuses almost exclusively on “character portrayal,” leaving the patterns for “translation quality” and “plot acceptance” underexplored.

Suggestion:
Refine the platform analysis: Consolidate the discussion on platform mechanisms and user bases into a more unified argument, framing the user base as a consequence of the platform’s design and primary function (e-commerce vs. social-literary).

Balance the plot acceptance discussion: While cultural hermeneutic gaps are certainly a factor, reframe the discussion to also emphasize the translational challenge this poses. The episodic structure represents a fundamental dilemma for the translator between faithfulness (foreignization) and readability (domestication). The negative sentiment is a valuable data point highlighting this enduring conflict, not just a measure of reader failure to understand.

Expand the translator-theme analysis: Provide succinct, strategy-based explanations for the observed differences in positive sentiment for each translator across all three themes. For example, briefly hypothesize why Yu's version scores highest on “translation quality” (likely due to scholarly apparatus) and why “plot acceptance” is consistently the most challenging theme for all translators.

**Response:** We thank the reviewer for these insightful suggestions. First, in response to the comment on Section 3.2, we have consolidated the discussion on platform differences by framing the user base as a consequence of the platform’s design and primary function, as suggested. The changes are in the revised manuscript (lines 427-454, pp. 21-22).

Comparison of Original and Revised Text

*Note: In the ‘Original’ column, strikethrough text indicates content that has been removed in the revised manuscript. In the ‘Revised’ column, text highlighted in red indicates new additions in the revised manuscript.*

| **Original** | **Revised** |
| --- | --- |
| ~~These divergences primarily stem from two factors. First, the fundamental contrast between Amazon’s e-commerce framework and Goodreads’ social reading ecosystem creates~~ ~~distinct evaluation mechanisms. On Amazon, its rating system is inherently tied to its e-commerce model, prioritizing purchase-related contexts that influence user evaluations. Reviews here frequently feature keywords like “buy,” “purchased,” and “price,” reflecting users’ focus on transactional aspects rather than literary content. This commercial orientation leads to more extreme ratings as users rate based on product satisfaction rather than textual merit [43].~~ ~~Conversely, Goodreads is designed as a social reading community, where users engage in detailed discussions about themes, writing styles, and character development. They treat ratings as a form of cultural capital, using them to perform literary expertise and participate in communal discourse, resulting in more nuanced, moderate ratings [44]. The platform’s algorithm also prioritizes review depth and engagement over transactional signals, reinforcing content-focused evaluations [45].~~    ~~Second,~~ ~~divergent reader bases critically shape distinct evaluation mechanisms between the platforms. Amazon’s user base is broader and more diverse, encompassing casual buyers who may rate books based on non-literary criteria. Reflecting this orientation, reviewers often submit short, utility-driven comments, with limited focus on literary analysis [46]. Conversely, Goodreads attracts self-identified core readers who actively engage with books as cultural artifacts. Within this ecosystem, users who frequently update reading statuses and participate in genre discussions demonstrate stronger emphasis on narrative complexity and thematic depth in evaluations [47]. Goodreads’ tagging system and community-driven “classics” categorization reflect a literate, critically engaged audience, whereas Amazon’s algorithmic recommendations cater to a mass market, skewing ratings toward accessibility and commercial appeal rather than literary merit [45].~~ | These divergent review patterns between Amazon and Goodreads can be traced to their distinct core functionalities, which fundamentally shape their respective user bases and, consequently, the evaluation mechanisms that emerge on each platform. Amazon’s framework is intrinsically built around its e-commerce model. This commercial orientation prioritizes purchase-related contexts, which in turn attracts a broad and diverse user base that includes many casual buyers [60]. These users often approach book reviews through the lens of product satisfaction, leading to evaluations based on non-literary criteria. This tendency is reflected in the frequent use of transactional keywords like “buy,” “purchased,” and “price” within reviews, and often results in shorter, more utility-driven comments with limited focus on literary analysis. The platform’s rating system, reinforcing this mindset, encourages more extreme ratings rather than nuanced assessments of textual merit [61].  In contrast, Goodreads is designed as a social-literary ecosystem centered on reading as a cultural practice [62]. This core functionality fosters a community of self-identified, engaged readers who interact with books as cultural artifacts. This user base is more likely to participate in genre discussions, update reading statuses, and use the platform’s tagging system and community-driven “classics” categorization [63]. Within this environment, ratings and reviews themselves become a form of cultural capital; users employ them to perform literary expertise and engage in communal discourse [64]. Consequently, this performative aspect of reviewing inherently cultivates more nuanced, moderate ratings and reviews that emphasize narrative complexity, thematic depth, and writing style.  Ultimately, the algorithmic underpinnings of each platform solidify these differences: Amazon’s recommendations cater to mass-market appeal and accessibility, while Goodreads’ algorithms prioritize review depth and community engagement [32]. Thus, the observed patterns in sentiment and evaluation are a direct consequence of the foundational design principles governing each space, as seen in the contrast between e-commerce and social-literary platforms. |

Full references for the citations added in the revised text are provided below.

32.	Murray S. Secret agents: Algorithmic culture, Goodreads and datafication of the contemporary book world. European Journal of Cultural Studies. 2021;24(4): 970–989. doi: 10.1177/1367549419886026

60.	Lee S, Ji H, Kim J, Park E. What books will be your bestseller? A machine learning approach with Amazon Kindle. EL. 2021;39(1): 137–151. doi: 10.1108/EL-08-2020-0234

61.	Dimitrov S, Zamal F, Piper A, Ruths D. Goodreads versus Amazon: The effect of decoupling book reviewing and book selling. In: Proceedings of the International AAAI Conference on Web and Social Media, vol. 9. 2015. pp. 602–605. doi: 10.1609/icwsm.v9i1.14662

62.	Albrechtslund AMB. Amazon, Kindle, and Goodreads: Implications for literary consumption in the digital age. Consumption Markets & Culture. 2020;23(6): 553–568. doi: 10.1080/10253866.2019.1640216

63.	Maity SK, Panigrahi A, Mukherjee A. Analyzing social book reading behavior on Goodreads and how it predicts Amazon best sellers. In: Kaya M, Alhajj R, editors. Influence and Behavior Analysis in Social Networks and Social Media. 1st ed. Cham: Springer International Publishing; 2019. pp. 211–235. doi: 10.1007/978-3-030-02592-2_11

64.	Burnet K. Producing cultural capital on social book platform Goodreads: The performativity of reading books through achievement and taste. Master’s thesis. Utrecht University, 2024.

Second, regarding the discussion of plot acceptance (Section 3.3.3), we have reframed it to better emphasize the translational dilemma between faithfulness and readability, rather than reader misinterpretation. This revision can be found in the manuscript (lines 634-650, pp. 30-31).

| **Original** | **Revised** |
| --- | --- |
| Second, the translated work’s fidelity to its original episodic structure also presents significant cross-cultural challenges. The original novel employs an episodic, cyclical structure known as Zhanghui Style, a chapter-linked narrative form that prioritizes moral coherence over plot novelty [57]. In *JTTW*, this episodic architecture is intrinsically connected to Buddhist philosophy, where each narrative cycle symbolizes sentient beings navigating ignorance towards wisdom [58]. The “eighty-one ordeals” mirror the Buddhist theory of karmic rebirth, where repetitive practices across lifetimes are essential for liberation [59]. This structure, far from a narrative flaw, serves as a “soteriological device” [60], dismantling layers of delusion until ultimate awakening. However, it inevitably clashes with Western expectations of linear, conflict-driven plotting [61]. ~~Readers accustomed to character arcs or escalating stakes often critique the repetition, as exemplified by~~: “…the story is long and can get repetitive at times…” ~~(marc11248); “The major takeaway I had from this book was how LONG and BORING the adventure was.~~ Once the main characters are established, they have encounter after encounter with trouble which inevitably goes as follows: 1) Monster sees Tang priest; 2) Monster kidnaps Tang priest; 3) Monkey saves the day. It is in fact so formulaic…” ~~(Nathaniel). These responses reflect both misinterpretations of the zhanghui form’s purpose and failure to recognize Buddhism’s structural role, exposing profound rifts between cultural hermeneutic frameworks.~~ | Second, the translated work’s fidelity to its original episodic structure also presents significant cross-cultural challenges. The original novel employs an episodic, cyclical structure known as Zhanghui Style, a chapter-linked narrative form that prioritizes moral coherence over plot novelty [74]. In *JTTW*, this episodic architecture is intrinsically connected to Buddhist philosophy, where each narrative cycle symbolizes sentient beings navigating ignorance towards wisdom [75]. The “eighty-one ordeals” mirror the Buddhist theory of karmic rebirth, where repetitive practices across lifetimes are essential for liberation [76]. This structure, far from a narrative flaw, serves as a “soteriological device” [77], dismantling layers of delusion until ultimate awakening. However, it inevitably clashes with Western expectations of linear, conflict-driven plotting [78]. Even in a translation celebrated for its scholarly rigor and fidelity，such as Yu’s edition, which receives the highest sentiment scores for translation quality, the positive sentiment for plot acceptance remains markedly lower (78.8%), as evidenced in Fig. 9. This clash is further reinforced by reader reviews, “…the story is long and can get repetitive at times…”; “Once the main characters are established, they have encounter after encounter with trouble which inevitably goes as follows: 1) Monster sees Tang priest; 2) Monster kidnaps Tang priest; 3) Monkey saves the day. It is in fact so formulaic…”.  While these reader critiques indeed stem from a clash of cultural hermeneutic frameworks, they also, and ultimately, reflect the translational challenges inherent in structural fidelity. More specifically, these responses are symptomatic of the inherent tension translators face between domesticating the text for target-reader fluency and foreignizing it to preserve cultural specificity, a core concern in translation studies [79]. For *JTTW*, this tension is acute. Remaining faithful to the soteriological function of the zhanghui form risks preserving a cyclical structure that feels repetitive to readers expecting linear plots. Conversely, prioritizing readability through streamlining risks eroding the Buddhist connotations and cultural essence that define the original. This ethical obligation to preserve cultural specificity is particularly critical for classical works, where simplification can reinforce ethnocentric biases [80].  Therefore, the elevated negative sentiment toward the plot should be interpreted not as a simple measure of reader misinterpretation, but as a valuable data point. It empirically captures the enduring conflict between cultural fidelity and reader accessibility inherent in translating a culturally embedded narrative. Thus, the reception of *JTTW*’s plot is shaped not only by cross-cultural hermeneutic gaps but, more profoundly, by the inherent and unavoidable dilemmas of the translation process itself. |

Full references for the citations added in the revised text are provided below.

79.	Venuti L. Translation changes everything: Theory and practice. 1st ed. London; New York: Routledge; 2013. doi: 10.4324/9780203074428

80.	Venuti L. The poet’s version; or, an ethics of translation. Translation Studies. 2011;4(2): 230–247. doi: 10.1080/14781700.2011.560021

Finally, we have expanded the analysis of Figure 9 to fully explore the patterns for “translation quality” and “plot acceptance.” For translation quality, we now explicitly demonstrate the sentiment variation across translators and propose an explanation for Yu's version scoring highest. The updated paragraph is located in the manuscript (lines 563-571, p. 27). For plot acceptance, we have conducted an interpretative analysis of the sentiment scores exhibited in Figure 9, embedding this data within a systematic analysis of why this theme poses the most significant challenge. These additions are located in the manuscript (lines 593-598, pp. 28-29; and lines 621-624, p. 30).

Regarding translation quality, the original and revised versions are presented below for comparison.

| **Original** | **Revised** |
| --- | --- |
| As evidenced in Fig 8, the semantic network reveals a densely interconnected lexical core manifesting a dual evaluative emphasis on literary reception and cultural mediation. Central descriptors “great” and “classic” signify readers’ strong approval of translational merit, while their convergence with “epic” further corroborates recognition of the novel’s canonical status. Simultaneously, the frequently co-occurred affect-laden terms like “recommend” reflect readers’ immersive engagement with the translated text and their endorsement of its literary and cultural value. Integral to this nexus, the words “know” and “learn” further reflect readers’ perceived acquisition of China-specific philosophical frameworks through the translated text. Adjacently, “culture” and “history” highlight the work’s efficacy as a cross-cultural conduit. Critically, bridging terms like “enlightenment” and “understand” denote successful epistemological transfer, mediating source-text tenets with target-reader cognition.  ~~The salient topic term “version” (Fig 8) underscores readers’ engagement with translational variants. Fig 9 reveals Yu’s translations received the highest proportion of positive evaluations, closely followed by Waley and Lovell, whereas Jenner recorded significantly fewer positive evaluations. This sentiment ranking partially corresponds to translator citation frequency in reviews: Waley (most cited, 118 times), Lovell (97), Yu (74), and Jenner (lowest, 21 times). Notably, Yu’s translations,~~ characterized by extensive paratextual elements, are consistently valued by readers as indispensable for contextual comprehension [54]. Despite occasional critiques of verbosity, these scholarly supplements bridge critical linguistic and cultural gaps while satisfying demands for authentic engagement with canonical Chinese literature [55]. This recognition is evidenced by recurring descriptive terms in reader reviews, such as “historical background” (ADobies) and “helpful background” (Emmanuel), alongside explicit reader affirmations including “…footnotes...necessary for complete understanding of the culture” (daoist1042). | As evidenced in Fig 8, the semantic network reveals a densely interconnected lexical core manifesting a dual evaluative emphasis on literary reception and cultural mediation. Central descriptors “great” and “classic” signify readers’ strong approval of translational merit, while their convergence with “epic” further corroborates recognition of the novel’s canonical status. Simultaneously, the frequently co-occurred affect-laden terms like “recommend” reflect readers’ immersive engagement with the translated text and their endorsement of its literary and cultural value. Integral to this nexus, the words “know” and “learn” further reflect readers’ perceived acquisition of China-specific philosophical frameworks through the translated text. Adjacently, “culture” and “history” highlight the work’s efficacy as a cross-cultural conduit. Critically, bridging terms like “enlightenment” and “understand” denote successful epistemological transfer, mediating source-text tenets with target-reader cognition.    The salient topic term “version” (Fig 8) underscores readers’ engagement with translational variants. To further quantify how readers evaluate these variants, Fig 9 provides translator-specific sentiment data for translation quality: Yu’s translations received the highest proportion of positive evaluations (89.6%), closely followed by Waley (86.9%) and Lovell (86.6%), whereas Jenner recorded significantly fewer positive evaluations (79.3%). This places Yu at the apex of the quality ranking, with a notable margin over the other translators. Beyond the quantitative findings, Yu’s preeminence in translation quality stems directly from his scholarly, foreignizing translation strategy. His work, characterized by extensive paratextual elements, is consistently valued by readers as indispensable for contextual comprehension [71]. Despite occasional critiques of verbosity, these scholarly supplements bridge critical linguistic and cultural gaps while satisfying demands for authentic engagement with canonical Chinese literature [72]. This recognition is evidenced by recurring descriptive terms in reader reviews, such as “historical background” and “helpful background”, alongside explicit reader affirmations including “…footnotes...necessary for complete understanding of the culture”. |

Regarding plot acceptance, the original and revised versions are presented below for comparison.

| **Original** | **Revised** |
| --- | --- |
| Nevertheless, comparative analysis in Fig 7 reveals plot acceptance’s negative sentiment rate (11.6%) surpasses other thematic dimensions, exceeding translation quality (5.9%) by 5.7 percentage points and character portrayal (9.5%) by 2.1 points. ~~This significant divergence correlates directly with reader reviews,~~ where terms like “long” (177 times) and “repetitive” (175) epitomize criticisms of narrative pacing and structure despite overall engagement.  A significant portion of these critiques stems from two interrelated factors. First…  Second, the translated work’s fidelity to its original episodic structure also presents significant cross-cultural challenges. The original novel employs an episodic, cyclical structure known as Zhanghui Style, a chapter-linked narrative form that prioritizes moral coherence over plot novelty [57]. In *JTTW*, this episodic architecture is intrinsically connected to Buddhist philosophy, where each narrative cycle symbolizes sentient beings navigating ignorance towards wisdom [58]. The “eighty-one ordeals” mirror the Buddhist theory of karmic rebirth, where repetitive practices across lifetimes are essential for liberation [59]. This structure, far from a narrative flaw, serves as a “soteriological device” [60], dismantling layers of delusion until ultimate awakening. However, it inevitably clashes with Western expectations of linear, conflict-driven plotting [61]. ~~Readers accustomed to character arcs or escalating stakes often critique the repetition, as exemplified by:~~ “…the story is long and can get repetitive at times…” ~~(marc11248); “The major takeaway I had from this book was how LONG and BORING the adventure was.~~ Once the main characters are established, they have encounter after encounter with trouble which inevitably goes as follows: 1) Monster sees Tang priest; 2) Monster kidnaps Tang priest; 3) Monkey saves the day. It is in fact so formulaic…” ~~(Nathaniel). These responses reflect both misinterpretations of the zhanghui form’s purpose and failure to recognize Buddhism’s structural role, exposing profound rifts between cultural hermeneutic frameworks.~~ | Nevertheless, comparative analysis in Fig 7 reveals plot acceptance’s negative sentiment rate (11.6%) surpasses other thematic dimensions, exceeding translation quality (5.9%) by 5.7 percentage points and character portrayal (9.5%) by 2.1 points. This significant divergence is further corroborated by the translator-level data in Fig 9, which shows that the positive sentiment proportion for plot acceptance clusters within a notably lower range (76.6% – 80.5%) across all translators, consistently trailing behind their respective scores for translation quality and character portrayal. This consistent pattern of challenge, evident at both the macro thematic level and the individual translator level, finds its direct manifestation in reader reviews, where terms like “long” (177 times) and “repetitive” (175) epitomize criticisms of narrative pacing and structure despite overall engagement.  A significant portion of these critiques stems from two interrelated factors. First…  Second, the translated work’s fidelity to its original episodic structure also presents significant cross-cultural challenges. The original novel employs an episodic, cyclical structure known as Zhanghui Style, a chapter-linked narrative form that prioritizes moral coherence over plot novelty [74]. In *JTTW*, this episodic architecture is intrinsically connected to Buddhist philosophy, where each narrative cycle symbolizes sentient beings navigating ignorance towards wisdom [75]. The “eighty-one ordeals” mirror the Buddhist theory of karmic rebirth, where repetitive practices across lifetimes are essential for liberation [76]. This structure, far from a narrative flaw, serves as a “soteriological device” [77], dismantling layers of delusion until ultimate awakening. However, it inevitably clashes with Western expectations of linear, conflict-driven plotting [78]. Even in a translation celebrated for its scholarly rigor and fidelity, such as Yu’s edition, which receives the highest sentiment scores for translation quality, the positive sentiment for plot acceptance remains markedly lower (78.8%), as evidenced in Fig. 9. This clash is further reinforced by reader reviews, “…the story is long and can get repetitive at times…”; “Once the main characters are established, they have encounter after encounter with trouble which inevitably goes as follows: 1) Monster sees Tang priest; 2) Monster kidnaps Tang priest; 3) Monkey saves the day. It is in fact so formulaic…”. |

**Comment 3**: 3. Enhancing Methodological Transparency and Scope
The methods are well-described, but some choices need further justification, and the scope could be more clearly bounded. The weighted fusion scheme for the general sentiment lexicons (NRC 0.4, AFINN 0.4, VADER 0.2) is presented without empirical justification. The choice of K=3 for the LDA model is well-argued based on coherence and interpretability, but the potential value of exploring a slightly higher K (e.g., K=4) to see if a meaningful sub-topic splits from one of the three main themes is not fully dismissed. Relying on two platforms, while practical, is a limitation that should be more explicitly acknowledged upfront.

Suggestion:
Justify the lexicon weights: Either provide a reference that empirically validates this specific weighting scheme for this type of text, or briefly describe a validation step (e.g., grid search on a annotated sample) used to arrive at these values. Alternatively, clearly state that the weighting was based on a theoretical assessment of each lexicon's relative strengths.

Acknowledge LDA trade-offs: A brief sentence acknowledging that while K=3 was optimal for high-level themes, future research with different parameters might uncover more nuanced sub-themes would strengthen the methodological reasoning.

State scope limitation clearly: Explicitly mention the focus on Amazon and Goodreads as a conscious delimitation of scope in the methods section, noting they represent two major but distinct types of reception spaces (commercial vs. social-literary). This turns a potential weakness into a justified methodological choice.

Overall, this is a strong piece of interdisciplinary research that makes a valuable contribution to the fields of translation studies, digital humanities, and cultural analytics. I am confident that addressing these above points will further elevate the quality of this impressive work.

**Response**: We thank the reviewer for these insightful methodological suggestions. First, in response to the comment on the lexicon weighting scheme, we have added a theoretical justification based on the relative strengths of each lexicon. This change is in the revised manuscript (lines 250-269, pp. 12-13).

Comparison of Original and Revised Text

*Note: In the ‘Original’ column, strikethrough text indicates content that has been removed in the revised manuscript. In the ‘Revised’ column, text highlighted in red indicates new additions in the revised manuscript.*

| **Original** | **Revised** |
| --- | --- |
| ~~Then, the lexicon fusion framework strategically integrates three general sentiment lexicons to complement the above domain sentiment lexicon. The NRC Emotion Lexicon was incorporated for its granular emotion taxonomy, enabling precise detection of reader responses to literary devices. AFINN enhances detection of colloquial sentiment expressions, effectively capturing informal evaluations unique to literary discourse. VADER supplements these through context-aware rules optimized for social media patterns.~~ ~~Following ensemble learning principles, a weighted fusion scheme assigns relative contributions of 0.4, 0.4, and 0.2 to NRC, AFINN, and VADER respectively [28]. This weighting reflects both the established efficacy of NRC and AFINN in broad sentiment analysis tasks and VADER’s potential domain adaptation limitations, thereby balancing generalizability with domain-specific precision through model diversity.~~ | The lexicon fusion framework strategically integrates three general sentiment lexicons to complement the domain-specific lexicon. The NRC Emotion Lexicon excels at capturing discrete, fine-grained emotions [38], making it well-suited for deciphering complex emotional expressions in literary reviews. Similarly, AFINN is distinguished by its high reliability in sentiment polarity judgment and ability to quantify emotional intensity [39], which aligns with the need to assess the strength of readers’ responses. VADER, optimized for informal short texts, demonstrates robustness in processing concise reviews [40], complementing the others in capturing context-sensitive cues. Rather than applying equal weights, which would overlook the lexicons’ heterogeneous contributions [41], we employed a weighted fusion scheme, assigning relative weights of 0.4, 0.4, and 0.2 to NRC, AFINN, and VADER, respectively. This weighting follows the established methodological principle that integrating multiple sentiment lexicons should be based on their adaptability to the target domain and their contribution to overall performance [42–44], an approach validated in prior lexicon construction studies [45]. NRC and AFINN were assigned higher weights due to their higher inherent adaptability to the stylistic and evaluative nature of literary reviews. Conversely, VADER, while valuable for informal language, received a lower weight owing to its primary optimization for social media contexts, which presents potential domain adaptation limitations for longer-form literary reviews. This configuration aims to balance generalizability with domain-specific precision by leveraging model diversity. |

Full references for the citations added in the revised text are provided below.

38.	Mohammad SM, Turney PD. Crowdsourcing a word–emotion association lexicon. Computational Intelligence. 2013;29(3): 436–465. doi: 10.1111/j.1467-8640.2012.00460.x

39.	Nielsen FÅ. A new ANEW: Evaluation of a word list for sentiment analysis in microblogs. arXiv.1103.2903 [Preprint]. 2011 [cited 2025 November 16]. Available from: http://arxiv.org/abs/1103.2903

40.	Hutto C, Gilbert E. VADER: A parsimonious rule-based model for sentiment analysis of social media text. In: Proceedings of the International AAAI Conference on Web and Social Media, vol. 8. 2014. pp. 216–225. doi: 10.1609/icwsm.v8i1.14550

41.	Cho H, Kim S, Lee J, Lee J-S. Data-driven integration of multiple sentiment dictionaries for lexicon-based sentiment classification of product reviews. Knowledge-Based Systems. 2014;71: 61–71. doi: 10.1016/j.knosys.2014.06.001

42.	Araque O, Zhu G, Iglesias CA. A semantic similarity-based perspective of affect lexicons for sentiment analysis. Knowledge-Based Systems. 2019;165: 346–359. doi: 10.1016/j.knosys.2018.12.005

43.	Wang L, Isomura S, Ptaszynski M, Dybala P, Urabe Y, Rzepka R, et al. The limits of words: Expanding a word-based emotion analysis system with multiple emotion dictionaries and the automatic extraction of emotive expressions. Applied Sciences. 2024;14(11): 4439. doi: 10.3390/app14114439

44.	Woudstra L, Dawodu M, Igwe F, Li S, Shi N, Hauer B, et al. Identifying emotional and polar concepts via synset translation. In: Proceedings of the 13th Joint Conference on Lexical and Computational Semantics (* SEM 2024). 2024. pp. 142–152.

45.	Vashishtha S, Susan S. Neuro-fuzzy network incorporating multiple lexicons for social sentiment analysis. Soft Comput. 2022;26(9): 4487–4507. doi: 10.1007/s00500-021-06528-0

Second, regarding the choice of K for the LDA model, we have added a sentence acknowledging the potential value of exploring higher values of K in future work. This revision is included in the manuscript (lines 319-322, pp. 15-16).

| **Original** | **Revised** |
| --- | --- |
| To further verify this domain-based insight and ensure the selected topic number K=3 also exhibited strong semantic coherence, we conducted a coherence score calculation using the *Cv* metric. The topic coherence evaluation revealed that K=3 demonstrated the second-highest score at 0.4356. Notably, this coherence score for K=3 exceeded that of K=4 (0.4280), suggesting better topic stability for three-topic modeling. Integrating both perplexity and coherence, we finalized K=3 as the optimal choice. This dual-metric approach for resolving perplexity ambiguities, ensures alignment with both statistical rigor [32] and domain-specific interpretability—essential for translation analysis where topic meaningfulness directly impacts downstream tasks [33]. | To further verify this domain-based insight and ensure the selected topic number K=3 also exhibited strong semantic coherence, we conducted a coherence score calculation using the $Cv$ metric. The topic coherence evaluation revealed that K=3 demonstrated the second-highest score at 0.4356. Notably, this coherence score for K=3 exceeded that of K=4 (0.4280), suggesting better topic stability for three-topic modeling. Integrating both perplexity and coherence, we finalized K=3 as the optimal choice. This dual-metric approach for resolving perplexity ambiguities, ensures alignment with both statistical rigor [49] and domain-specific interpretability—essential for translation analysis where topic meaningfulness directly impacts downstream tasks [50]. It is noteworthy that while K=3 was determined to be optimal for identifying the high-level thematic structure central to our research questions, it should be acknowledged that a slightly higher K (e.g., K=4) still has the potential to uncover more nuanced subthemes. |

Finally, concerning the study’s scope, we have added a statement that explicitly frames the focus on Amazon and Goodreads as a deliberate choice, highlighting their distinct reception spaces. This addition can be found in the manuscript (lines 195-204, pp. 9-10).

| **Original** | **Revised** |
| --- | --- |
| We selected reviews of the four most-reviewed *JTTW* translations—by Arthur Waley, Anthony C. Yu, Julia Lovell, and W.J.F. Jenner—as research data. This approach, preferable to single-translator analysis or unfiltered keyword searches (e.g., “Journey to the West”), addresses three fundamental constraints. First, it ensures substantial data for computationally intensive methods such as LDA topic modeling and sentiment analysis as individual translators’ works typically attract insufficient review counts. Second, it enables examination of translational styles and platform-mediated reception dynamics, core dimensions in reception studies. Third, it mitigates data noise by excluding irrelevant responses inherent in indiscriminate collection. | We selected reviews of the four most-reviewed *JTTW* translations—by Arthur Waley, Anthony C. Yu, Julia Lovell, and W.J.F. Jenner—as research data. This approach, preferable to single-translator analysis or unfiltered keyword searches (e.g., “Journey to the West”), addresses three fundamental constraints. First, it ensures substantial data for computationally intensive methods such as LDA topic modeling and sentiment analysis as individual translators’ works typically attract insufficient review counts. Second, it enables examination of translational styles and platform-mediated reception dynamics, core dimensions in reception studies. Third, it mitigates data noise by excluding irrelevant responses inherent in indiscriminate collection.  Having defined the corpus through translator selection, we deliberately delimited the data sources to two major online platforms, Amazon and Goodreads. This selection is justified by their distinct and complementary positions within the literary ecosystem. Goodreads, described as “the Anglophone world’s dominant book-centric social networking platform” [32], specializes in community-driven discourse exploring textual nuances and reading experiences [33]. Conversely, Amazon, as a premier commercial retailer, generates user reviews focused on practical, purchase-related evaluations[34]. This strategic focus on two platform types—social-literary and commercial—ensures the analysis captures a broad spectrum of reader responses, from literary evaluation to utilitarian judgment. |

Full references for the citations added in the revised text are provided below.

32.	Murray S. Secret agents: Algorithmic culture, Goodreads and datafication of the contemporary book world. European Journal of Cultural Studies. 2021;24(4): 970–989. doi: 10.1177/1367549419886026

33.	Singson M. Examining the relationship between academic book citations and Goodreads reader opinion and rating. ALIS. 2021;67(4): 215–221. doi: 10.56042/alis.v67i4.32597

34.	Zhang C, Tong T, Bu Y. Examining differences among book reviews from various online platforms. OIR. 2019;43(7): 1169–1187. doi: 10.1108/OIR-01-2019-0037.

**Response to Reviewer #3**

We sincerely thank the reviewer for the thorough understanding and exceptionally positive assessment of our work. We truly appreciate the depth and generosity of their comments. In response to the specific and encouraging points raised, we provide the following.

**Comment**: The core highlight of the paper lies in the innovation and adaptability of its methodology. Addressing issues such as insufficient domain adaptability and contextual misjudgment of general sentiment dictionaries in literary translation analysis, the researchers constructed a hybrid sentiment dictionary integrating domain-specific sentiment dictionaries with AFINN, NRC, and VADER. Through mechanisms like weighted integration, negation handling, and transition tone attenuation, it effectively resolves the semantic shift issue of words such as "literal" and "rebellious" in translation reviews. Meanwhile, combining LDA topic modeling, three core themes (translation quality, plot acceptability, and characterization) were identified under the dual verification of statistical indicators and domain interpretability. This achieves the organic unity of quantitative analysis and humanistic interpretation, breaking through the limitations of traditional translation reception studies—dominated by qualitative analysis and with limited sample sizes.

The limitations of the paper are also worthy of attention. The study covers only two English-language platforms, and the cultural representativeness of the samples needs to be expanded. Although the domain-specific sentiment dictionary has undergone double-expert annotation, it still requires larger-scale collaborative annotation for further improvement. However, the researchers have clearly proposed prospects for expanding the platform scope and optimizing dictionary adaptability in the future, leaving a clear path for subsequent studies.

Overall, taking the English translations of Journey to the West as a sample, this paper systematically depicts the complex landscape of the overseas reception of Chinese classical literature through innovations in digital humanities methods. Its triple analytical framework not only provides a reusable methodological template for translation reception studies but also deepens the interpretation of cross-cultural communication theory in the digital age by revealing the interactive relationship between platform mechanisms, translators' strategies, and readers' cognition. Against the backdrop of culture communication, such studies integrating technical tools with humanistic care undoubtedly offer important academic reference and practical enlightenment for the overseas communication and reception evaluation of classical literature.

**Response:** We are profoundly grateful to the reviewer for the truly generous and insightful assessment of our work. We are truly encouraged that the reviewer recognizes the core contributions we aimed to make, particularly in methodological innovation and the integration of computational analysis with humanistic interpretation. We also deeply appreciate your understanding of the study's significance for translation reception studies and cross-cultural communication. Such positive and constructive feedback is invaluable and will significantly motivate our future research in this area.

Once again, we sincerely thank the editors and the reviewers for their time and constructive comments, which have significantly strengthened our manuscript. We believe we have addressed all points raised, and we hope the revised version now meets the journal's standards. We look forward to your decision and are happy to provide any further information if needed.

Best regards,

Ningning Jia,

Corresponding Author

E-mail: nnjia_ac@163.com
